# Paternal malnutrition programs breast cancer risk and tumor metabolism in offspring

**DOI:** 10.1186/s13058-018-1034-7

**Published:** 2018-08-30

**Authors:** Raquel Santana da Cruz, Elissa J. Carney, Johan Clarke, Hong Cao, M. Idalia Cruz, Carlos Benitez, Lu Jin, Yi Fu, Zuolin Cheng, Yue Wang, Sonia de Assis

**Affiliations:** 1 Department of Oncology, Lombardi Comprehensive Cancer Center, Georgetown University, 3970 Reservoir Road, NW, The Research Building, Room E410, Washington, DC, 20057 USA; 2 The Bradley Department of Electrical and Computer Engineering, Virginia Polytechnic Institute and State University Research Center, Arlington, VA USA

**Keywords:** Ancestral nutrition, Paternal programming, Breast cancer, AMPK pathway

## Abstract

**Background:**

While many studies have shown that maternal factors in pregnancy affect the cancer risk for offspring, few studies have investigated the impact of paternal exposures on their progeny’s risk of this disease. Population studies generally show a U-shaped association between birthweight and breast cancer risk, with both high and low birthweight increasing the risk compared with average birthweight. Here, we investigated whether paternal malnutrition would modulate the birthweight and later breast cancer risk of daughters.

**Methods:**

Male mice were fed AIN93G-based diets containing either 17.7% (control) or 8.9% (low-protein (LP)) energy from protein from 3 to 10 weeks of age. Males on either group were mated to females raised on a control diet. Female offspring from control and LP fathers were treated with 7,12-dimethylbenz[a]anthracene (DMBA) to initiate mammary carcinogenesis. Mature sperm from fathers and mammary tissue and tumors from female offspring were used for epigenetic and other molecular analyses.

**Results:**

We found that paternal malnutrition reduces the birthweight of daughters and leads to epigenetic and metabolic reprogramming of their mammary tissue and tumors. Daughters of LP fathers have higher rates of mammary cancer, with tumors arising earlier and growing faster than in controls. The energy sensor, the AMP-activated protein kinase (AMPK) pathway, is suppressed in both mammary glands and tumors of LP daughters, with consequent activation of mammalian target of rapamycin (mTOR) signaling. Furthermore, LP mammary tumors show altered amino-acid metabolism with increased glutamine utilization. These changes are linked to alterations in noncoding RNAs regulating those pathways in mammary glands and tumors. Importantly, we detect alterations in some of the same microRNAs/target genes found in our animal model in breast tumors of women from populations where low birthweight is prevalent.

**Conclusions:**

Our study suggests that ancestral paternal malnutrition plays a role in programming offspring cancer risk and phenotype by likely providing a metabolic advantage to cancer cells.

**Electronic supplementary material:**

The online version of this article (10.1186/s13058-018-1034-7) contains supplementary material, which is available to authorized users.

## Background

Parental environmental exposures have been shown to affect phenotype in the next generation [1]. Given the close relationship between mother and the fetus, most of the evidence for this phenomenon comes from maternal exposures in pregnancy. However, many recent studies both in animals and humans have shown that paternal environmental exposures ranging from stress to nutrition can influence the risk of disease in the offspring. Paternal ancestral exposures have been shown to affect organ development in offspring including brain, liver, pancreas, bone, and mammary tissue [2–6]. Many of these studies also suggest that memory of past paternal exposures is transmitted to the progeny through the germline via epigenetic mechanisms. More recently, it was reported that the noncoding RNA sperm load, particularly microRNAs (miRNAs) and tRNA-derived fragments (tRFs), plays an essential role in transmitting environmental memory from fathers to offspring [7–9] .

Epidemiologic and animal studies have consistently linked birthweight to cancer risk, particularly breast cancer [10–13]. Population studies generally show a U-shaped association between birthweight and breast cancer risk, with both high and low birthweight increasing risk compared with average birthweight [11, 13]. In these studies, the child’s birthweight is often attributed to maternal weight gain and nutrition in pregnancy. However, paternal factors also play a role in the offspring’s birthweight [14]. In line with this, we recently reported that paternal high-fat diet intake or overweight leads to increased birthweight, alterations in mammary gland development, and higher rates of breast cancer risk in daughters in two rodent models [5, 6]. While no studies in humans have directly investigated the link between paternal nutrition and breast cancer in daughters, an association between other paternal factors and cancer risk in the progeny has been reported [15–17].

Moderate paternal malnutrition such as protein restriction has been shown to impact the development of the offspring’s organs and metabolism [3, 4, 7], yet its influence on mammary development is unknown. Studies in humans suggest that this may be the case as women who were conceived during a famine have increased breast cancer risk [18]. Here, we investigated whether paternal suboptimal nutrition (low-protein (LP) diet) can modulate the birthweight, mammary development, and breast cancer risk of daughters using a mouse model. We found that LP daughters have lower birthweight, alterations in mammary gland morphology, and higher rates of mammary cancer. Furthermore, we found that mammary glands and tumors of LP daughters are metabolically rewired, with alterations in the AMP-activated protein kinase (AMPK) and amino-acid metabolism pathways. These changes were associated with differential expression of miR-451a, miR-200c, and miR-92a. Importantly, some of the same miRNAs and target gene alterations were detected in breast tumors of women from populations with high rates of a low birthweight.

## Methods

### Breeding and dietary exposures

The c57bl/6 strain of mice was used in all experiments. Male mice were fed AIN93G-based diets (Additional file 1: Table S1) manufactured by Envigo Teklad Diets (Madison, Wisconsin, USA) containing either 17.7% (control, *n* = 11) or 8.9% energy from protein (LP, *n* = 11) starting after weaning (3 weeks of age). Male bodyweight was recorded weekly. At 10 weeks of age, LP-fed and control-fed male mice were mated to female mice reared on the control diet to generate the female offspring (Additional file 1: Figure S1). Males were kept in female cages for 3 days. Female mice were kept on the control diet during the breeding period, for the extent of pregnancy (21 days), and after giving birth. The birthweight of pups and number of pups per litter was determined. To avoid a litter effect, pups were cross-fostered 2 days after dams gave birth. Pups from 2 to 3 dams were pooled and housed in a litter of 8–10 pups per nursing dam. All pups were weaned on postnatal day 21 and fed the control diet throughout the experiment. Pup bodyweight was recorded weekly. The female offspring of control or LP fathers were used to study birthweight, mammary gland morphology, molecular analyses, and mammary tumorigenesis as described in the following sections. The number of contributing fathers and female offspring used in each experiment is listed in Additional file 1 (Table S2). All animal procedures were approved by the Georgetown University Animal Care and Use Committee, and the experiments were performed following the National Institutes of Health guidelines for the proper and humane use of animals in biomedical research.

### Mature spermatozoa collection and purification

Control and LP fathers were euthanized once mating was completed and the caudal epididymis (for sperm collection) was dissected. The cauda and vas deferens from male mice were collected, punctured, and transferred to a tissue culture dish containing M2 media (M2 medium with HEPES, without penicillin and streptomycin, liquid, sterile-filtered, and suitable for mouse embryo; SIGMA, product #M7167) where it was incubated for 1 h at 37 °C. Sperm samples were isolated and purified from somatic cells. Briefly, the samples were washed with phosphate-buffered saline (PBS), and then incubated with somatic cell lysis buffer (SCLB; 0.1% SDS, 0.5% TX-100 in diethylpyrocarbonate water) for 1 h. SCLB was rinsed off with two washes of PBS and the purified spermatozoa sample was pelleted and used for DNA and miRNA extraction. To further rule out any somatic cell contamination, quantitative polymerase chain reaction (qPCR) was performed using RNA from purified sperm samples and primers for the somatic cell marker *Actb*, or the sperm-specific marker *Smcp* (Additional file 1: Figure S2).

### Bodyweight monitoring

The bodyweight of female offspring was measured at birth (*n* = 10–12 litters/group) and weekly after weaning (*n* = 29–32/group). Birthweights were analyzed by *t* test, and longitudinal bodyweight analyzed using two-way analysis of variance (ANOVA; group and time), with Sidak’s multicomparison test used for post-hoc analyses.

### Mammary gland harvesting

Inguinal mammary glands (fourth pair) of the female offspring of control and LP fathers (*n* = 8–10/group) were collected on postnatal day (PND) 50 and used for mammary gland development analysis, DNA, RNA, and protein extraction.

### Small RNA-seq analysis

Total RNA (small and large transcripts) was isolated from paternal sperm and the mammary tissue of offspring (*n* = 4/group/tissue) using Qiagen’s miRNeasy extraction kit according to the manufacturer’s instructions. Small noncoding RNA transcript libraries were constructed according to the Illumina TrueSeq Small RNA Pre-Kit. Indexed, paired-end sequencing libraries were prepared from quality total RNA (RIN ≥ 8). For each library, 10 million reads (raw data) were generated by Illumina Hi-Seq 4000. The raw reads were subject to adapter trimming and low-quality filtering using the Trimmomatic program. The high-quality clean reads were aligned on the mouse genome. Noncoding RNA tags were mapped to the mouse genome (GRCm38/mm10 reference genome) to analyze their expression and distribution on the genome. Small RNA tags were annotated with miRNA, tRNA, piRNA, and rRNA to miRBase, Ref-seq, GenBank, and Rfam databases using blastn with standard parameters. To analyze the differential expression of small RNA between control and LP groups, different small RNA species (miRNA, tRF, etc.) were normalized to TPM (transcripts per kilobase million). When needed, cross-sample re-normalization and/or batch-effect correction were performed [19]. The *P* value and *q* value between groups was generated using model-based and/or permutation-based significance tests. Small RNA with a *q* value less than 0.05 were considered significant, with an appropriate correction for multiple testing [19]. Target prediction for microRNAs of interest was conducted using TargetScan (Release 6.2). The predicted targeted mRNA list was then uploaded to ingenuity pathway analysis (IPA) for gene set enrichment analysis.

### Quantitative real-time PCR validation (qRT-PCR)

Differentially methylated genes (mammary tissue only) or differentially expressed miRNA (mammary tissue/tumors) uncovered by our bioinformatics team were validated in mammary tissues (*n* = 7–10/group) and tumors (*n* = 12/group) using qRT-PCR. Briefly, cDNA was synthesized from total RNA samples using the TaqMan™ Advanced miRNA cDNA Synthesis Kit (Applied Biosystems). PCR products were amplified from cDNA using TaqMan® Fast Advanced Master Mix and sequence-specific primers from TaqMan Assays (Applied Biosystems). Fold-change was calculated from Ct values and the expression levels of specific miRNAs were determined by normalizing these values with the fold-change values for appropriate endogenous controls (miR-361 or miR-26A for miRNAs and GAPDH for genes). Fold-differences between the groups were analyzed using a *t* test.

### Genome-wide DNA methylation analysis

Genomic DNA was isolated from paternal sperm and the mammary tissue of offspring (*n* = 2–3/group/tissue) using Qiagen’s DNeasy extraction kit according to the manufacturer’s instructions. Methylated DNA was eluted by the MethylMiner Methylated DNA Enrichment Kit (Invitrogen). Briefly, 1 μg of genomic DNA was sheared by sonication and captured by MBD proteins. The methylated DNA was eluted and used to generate methyl-CpG binding domain-based capture (MBDCap) libraries as previously described [20]. MBDCap coupled with massively parallel sequencing (MBDCap-seq) libraries were sequenced using the Illumina Genome Analyzer II (GA II). Image analysis and base calling were performed with the standard Illumina pipeline. Sequencing reads was mapped by the ELAND algorithm.

### Data analysis

The gene promoter regions are defined as up to 5000 base pairs upstream of the transcription starting site, as described in our previous study [20], and also included the first exon, since methylation in these two regions most strongly correlate with reduced gene expression [21–23]. We used a novel statistical approach, AISAIC [20, 24, 25], to determine the significant ‘driver’ methylation changes between LP and control offspring. To detect significant methylation changes (against background random events), methylation intensity fold-change is used as the differential methylation measure. The total number of bases of short reads in a promoter representing regional methylation intensity are used for calculating the fold-change, the *j*th promoter in the *i*th generation, i.e., f_*ij*_ = (Icase,_*ij*_ + βL)/(I_*control,ij*_ + βL), where I_*case,ij*_ and I_*control,ij*_ are the methylation intensities in case and control samples, respectively. L (= 36) is the short-read length in MDBCap-seq data, and β (= 10) is an offset parameter that suppresses the effects of weak methylation signals in the denominator [20]. Due to potential imbalanced rates of hypermethylation and hypomethylation, the null distribution of M_*j*_ can be asymmetric in some chromosomes. Thus, we adopted a one-sided test to separately evaluate the significance (*p* < α0 after multiple testing correction) of hypermethylation or hypomethylation. To address the complex ‘length’-dependent background rates, we exploited M_*j*_ score on flexible intervals, i.e., promoters, CpG islands, and CpG sites, to account for the potentially correlated effect by nearby CpG sites. We exploited the intrinsic correlation among nearby CpG sites and calculated and assigned an M-score to each methylation unit. Over the samples, we first merged nearby CpG sites into methylation intervals with flexible lengths, leaving the gaps of no/minimal CpG sites; then we divided each methylation interval into methylation units by the ‘break points’ of minimal correlation between CpG sites. Intuitively, a methylation unit contains a subset of highly correlated nearby CpG sites. In addition to permuting the promoter, CpG island, or CpG site, we permutated methylation units to account for different background rates of methylation units with varying lengths. Accordingly, we assessed the significance of length-specific methylation units, against the null hypothesis, similar to Algorithm 1 in Yuan et al. [24]. To address the biased null distribution problem, we iteratively detected significant methylation events and re-estimated an unbiased null distribution by an events-exclusive permutation scheme. This step improves the detection sensitivity Theorem 1 in Yuan et al. because conventional permutation schemes cannot distinguish between the contributions of sporadic methylations (obeying the null distribution) and true methylations (deviating from null distribution) to estimating null distributions, resulting in a theoretically conservative estimate [24].

### Mammary gland development

Inguinal mammary glands (fourth pair, *n* = 8–9/group) were stretched onto a slide, placed in a fixative solution, and stained with a carmine aluminum solution (Sigma Chemical Co.) as previously described [26]. Whole mounts were examined under the microscope and ductal elongation and number of terminal end buds (TEBs; undifferentiated structure considered to be the targets of malignant transformation), as previously described [26]. In addition, whole-mount images were analyzed using the ImageJ software (National Institute of Health, Bethesda, MD, USA). The area and integrated density of a manually drawn perimeter around the mammary epithelial tree of each whole-mount were measured. The average area as well as integrated density measurements are reported for each group. Differences were statistically tested using a *t* test (or corresponding nonparametric test).

### Mammary tumorigenesis

Mammary tumors were induced in female mice offspring (*n* = 29–32/group) by administration of medroxyprogesterone acetate (MPA; 15 mg, subcutaneously) at 6 weeks of age, followed by three weekly doses of 1 mg 7,12-dimethylbenz[a]anthracene (DMBA; Sigma, St. Louis, MO) dissolved in peanut oil by oral gavage. This established model of breast cancer has been used by us and others [5, 27]. Mice were examined for mammary tumors by palpation once per week, starting at week 2 after the last dose of DMBA and continue for a total of 20 weeks. Tumor growth was measured using a caliper, and the width and height of each tumor were recorded. The endpoints for data analysis were: i) latency to tumor appearance; ii) the number of animals with tumors (tumor incidence); and iii) the number of tumors per animal (tumor multiplicity). During follow-up, those animals in which tumor burden approximated 10% of total bodyweight were sacrificed, as required by our institution. Histopathology of tumors was evaluated commercially by Animal Reference Pathology (Salt Lake City, Utah). Differences in tumor latency and multiplicity were analyzed by a *t* test (or corresponding nonparametric test). Kaplan-Meier survival curves were used to compare differences in tumor incidence, followed by the log-rank test. Tumor growth was analyzed using two-way ANOVA (group and time), with Sidak’s multicomparison test used for post-hoc analyses.

### Analysis of cell proliferation

Cell proliferation was evaluated in PND50 inguinal mammary glands (fourth pair, *n* = 6/group) and mammary tumors (*n* = 6/group) by Ki-67 immunohistochemistry. Briefly, tissues were fixed in 10% buffered formalin, embedded in paraffin, and sectioned (5 μm). Sections were deparaffinized with xylene and rehydrated through a graded alcohol series. Antigen retrieval was performed by immersing the tissue sections at 98 °C for 40 min in 1× Diva Decloaker (Biocare). Tissue sections were treated with 3% hydrogen peroxide and 10% normal goat serum for 10 min, and were incubated with the primary antibody (Additional file 1: Table S3) overnight at 4 °C. After several washes, sections were treated to the appropriate horseradish peroxidase (HRP)-labeled polymer for 30 min and DAB chromagen (Dako) for 5 min. Slides were counterstained with hematoxylin (Fisher, Harris Modified Hematoxylin), blued in 1% ammonium hydroxide, dehydrated, and mounted with Acrymount. The sections were photographed using an Olympus IX-71 Inverted Epifluorescence microscope at 20× magnification. The proliferation index was determined by calculating the percentage of Ki67-positive cells among 1000 cells per slides. Images were evaluated with ImageJ software (National Institute of Health, Bethesda, MD, USA). Results were analyzed by *t* test (or corresponding nonparametric test).

### Analysis of cell apoptosis

Cell apoptosis analysis was conducted in PND50 inguinal mammary glands (fourth pair, *n* = 7–9/group) and mammary tumors (*n* = 10/group) by morphological detection. Tissues were fixed in neutral-buffered 10% formalin and stained with hematoxylin and eosin (H&E). Cells presenting loss of adhesion between adjacent cells, cytoplasmic condensation, and formation of apoptotic bodies were considered apoptotic. The sections were photographed using a Nikon E600 Epifluorescence microscope at 100× magnification. The apoptotic index was determined by the percentage of apoptotic bodies among 1000 cells per slide. Images were evaluated with ImageJ software (NIH, USA). Results were analyzed by *t* test (or corresponding nonparametric test).

### Analysis of protein levels in LP and control offspring mammary tissues and mammary tumors

Protein levels were assessed by Western blot in mammary tissues (*n* = 4–7/group) and tumors obtained from LP or control female offspring (*n* = 5/group). Total protein was extracted from mammary tissues using RIPA buffer with Halt™ Protease Inhibitor Cocktail (Thermo Fisher). Protein extracts (10 μg) were resolved on a 4–12% denaturing polyacrylamide gel (SDS-PAGE). Proteins were transferred using the iBlot® 7-Minute Blotting System (Invitrogen, USA) and blocked with 5% nonfat dry milk for 1 h at room temperature. Membranes were incubated with the specific primary antibodies (for antibody specifications and dilutions, see Additional file 1: Table S3) at 4 °C overnight. After several washes, the membranes were incubated with HRP-conjugated secondary antibody at room temperature for 1 h. Membranes were developed using the Chemiluminescent HRP antibody detection reagent (Denville Scientific Inc., USA), and exposed to blot imaging systems (Amersham™ Imager 600, GE Healthcare Life Sciences). Optical density of the bands was quantified using Quantity-one software (BIO-RAD, USA). To control for equal protein loading, expression of the proteins of interest was normalized to the β-actin or β-tubulin signal.

### Amino-acid analysis in LP and control offspring mammary tissues and mammary tumors

Tumor amino acid levels were measured using liquid chromatography/mass spectrometry (LC/MS; *n* = 6/group). Briefly, tissues were kept on ice for thawing before the extraction procedure. At total of 200 μL methanol was added prior to homogenization and 10 μl of the internal standard was added to these vials; 10 μL of calibrant standards and QC solution was then added to the respective vials. The derivatizing reagent was prepared by mixing 950 μL of water, ethanol, and pyridine along with 150 μL of phenyl isothiocyanate; 50 μL of this mixture was then added to the vials and left at room temperature for 20 min. The excess liquid was removed from the vials by drying under nitrogen for 60 min and 300 μL of 5 mM ammonium acetate in methanol was added to the vials and kept on the shaker for 15 min. The vials were centrifuged at 13,000 rpm for 10 min at 4 °C and the supernatant transferred to mass spectrometry vials. Then 5 μL of the samples were injected onto a Waters Acquity UPLC- Xevo TQ-S system (Waters Corporation, Milford, USA). Levels of glutamine were validated in tumors (*n* = 5/group) and measured in normal mammary tissue (*n* = 5–7/group) and plasma (*n* = 5–6/group) using a colorimetric assay kit (BioVision, CA). Levels of glutamate were measured in tumors, mammary tissue, and plasma using a colorimetric assay kit (BioVision, USA). For both assays, samples were diluted in assay buffer and placed into a 96-well plate and the protocol provided by the manufacturer was then followed. Absorbance was measured at 450 nm. The concentrations of glutamine and glutamate were calculated according to their respective standard curves. LC/MS results were statistically analyzed using two-way ANOVA (group and amino-acid type), with Sidak’s multicomparison test used for post-hoc analyses. Colorimetric assay results were analyzed using two-way ANOVA (group and tissue type), with Tukey’s test used for post-hoc analyses.

### TCGA analysis

The Cancer Genome Atlas (TCGA) database (http://cancergenome.nih.gov/) is classified by data type and data level to allow structured access to this resource with appropriate patient privacy protection. In this study, level 3 normalized gene level miRNA and mRNA next-generation sequencing data and corresponding demographic and clinical information for 1098 breast cancer patients were obtained from TCGA. Statistical analysis was conducted using DESeq2 package in R. Two kinds of comparison were made: one based on race/ethnicity, the other based on estrogen receptor (ER), progesterone receptor (PR), and human epidermal growth factor receptor (HER)2 status.

## Results

### Low protein consumption has no effect on paternal bodyweight but alters sperm epigenetic profile

Male mice (c57bl/6) consumed either a control or low-protein (LP) diet from 3 to 10 weeks of age (experimental design; Additional file 1: Figure S1). No difference in bodyweight gain was observed in males consuming a LP diet compared with controls (*P* = 0.99; Additional file 1: Figure S3a).

We and others have shown that the male germline can be epigenetically reprogrammed due to lifestyle and environmental exposures [2, 3, 5, 6, 9, 28]. Thus, we performed RNA-seq and MBD-seq analyses to assess small RNA and DNA methylation patterns, respectively, in mature sperm of control and LP males. Compared with controls, sperm from LP males showed several differentially methylated genes (promoter and exon1 regions). Of these, 14 were hypomethylated and 10 were hypermethylated (Additional file 1: Figure S3b).

The two groups had similar abundance and distribution of the major small RNA species. In agreement with previous reports [7, 29], tRFs were the most abundant sperm small RNA subtype, followed by miRNAs and piRNAs (Additional file 1: Figure S3c, d and Table S4). A total of 16 miRNAs were differentially expressed, with eight down- and eight upregulated, in sperm of LP mice compared with controls (Additional file 1: Figure S3e). In addition, a significant increase was observed in the expression of tRF5-Ile-TAT, tRF5-Arg-ACG, and tRF5-SeC-TCA, while tRF5-Pro-AGG and tRF5-Ser-CGA were significantly decreased in LP sperm compared with controls (Additional file 1: Figure S3f).

### LP female offspring have decreased birthweight and increased breast cancer risk

**Fig. 1:**
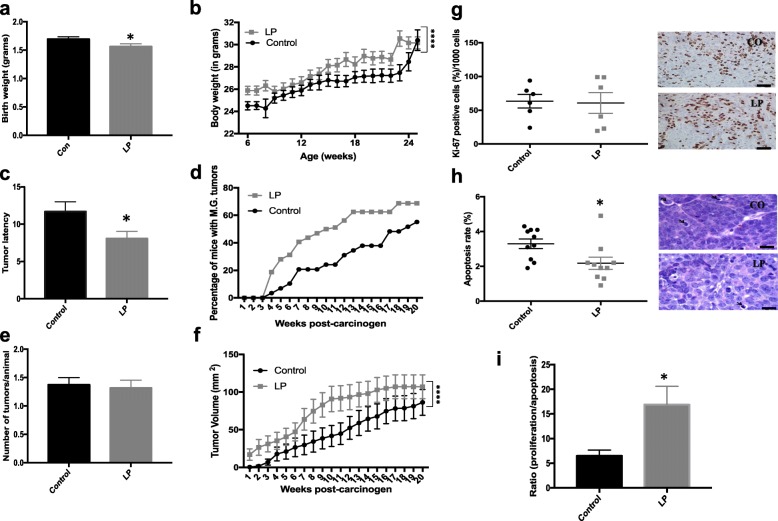
Paternal low-protein (LP) diet modulates birthweight and breast cancer risk in female offspring. Body weight (mean ± SEM) of LP female offspring **a** at birth and **b** longitudinally. **c** Mammary tumor latency (mean ± SEM), **d** mammary tumor incidence, **e** mammary tumor multiplicity (mean ± SEM), and **f** mammary tumor growth (mean ± SEM) in control (Con) and LP female offspring (*n* = 29–32/group). **g** Proliferation staining (Ki-67) quantification (percentage/1000 cells) with representative control and LP mammary tumor sections (20× magnification, mean ± SEM, *n* = 6/group), **h** morphological quantification (percentage/1000 cells) of apoptotic cells with representative control and LP mammary tumor sections (100× magnification, mean ± SEM, *n* = 10/group), and **i** proliferation/apoptosis ratio in control and LP mammary tumors. Significant differences versus the control group were determined as follows: *t* test (birthweight, tumor multiplicity and latency, cell proliferation, and apoptosis), log-rank test (tumor incidence), and two-way ANOVA (longitudinal bodyweight and tumor growth). *P < 0.05, ****P < 0.0001. Scale bars = 20 μm (**g**,**h**)

Next, we examined the offspring of control or LP male mice mated with female mice reared on the control diet. No differences in gender distribution were observed between litters from control and LP fathers (Additional file 1: Figure S4). Paternal LP consumption was associated with decreased birthweight of female (*P* = 0.035; Fig. 1a) but not male offspring (*P* = 0.45, data not shown). The decrease in the bodyweight of female offspring persisted through the prepubertal period when LP offspring began to gain more weight than controls animals, with LP females being slightly but significantly heavier over a 24-week monitoring period (*P* < 0.0001; Fig. 1b) compared with controls.

Low birthweight has been associated with higher risk for breast cancer in humans [11, 13] and animal models [30]. Thus, we studied the mammary cancer risk in control and LP daughters using a well-established [5, 27] carcinogen-induced mouse model of breast cancer. Tumor latency was significantly shorter in LP daughters compared with controls (*P* = 0.029; Fig. 1c). The incidence of palpable mammary tumors in the LP female mice was higher than in controls but did not reach statistical significance (*P* = 0.1; Fig. 1d). Tumor multiplicity was not different between the groups (*P* = 0.77; Fig. 1e). However, tumor growth was significantly increased in LP daughters at several time points (*P* < 0.0001; Fig. 1f). All mammary tumors included in our analyses were classified as mammary carcinomas by a pathologist.

Because we observed decreased mammary tumor latency and increased tumor growth in the LP offspring, we assessed cell proliferation and apoptosis rates in their tumors. We did not find differences in rates of cell proliferation (*P* = 0.89; Fig. 1g) between the groups, but detected a significant decrease in apoptosis (*P* = 0.023; Fig. 1h) in mammary tumors of LP offspring compared with controls. Consequently, the proliferation/apoptosis ratio was increased in LP tumors compared with controls (*P* = 0.018; Fig. 1i).

### Mammary gland development and epigenome of LP daughters are altered

**Fig. 2:**
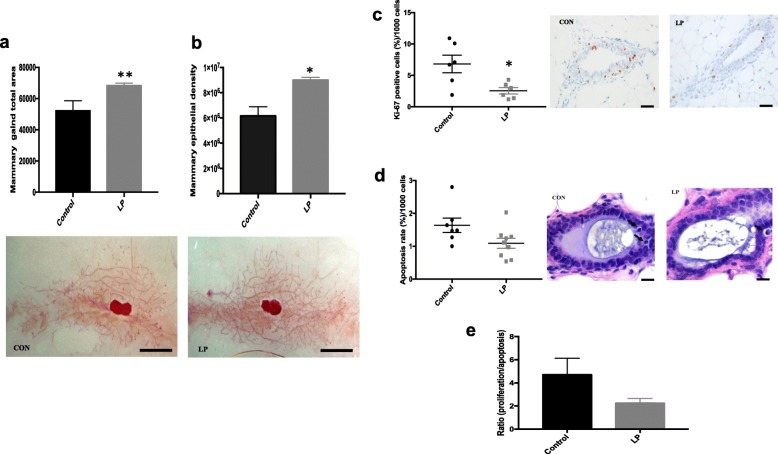
Paternal low-protein (LP) diet programs the mammary gland development of female offspring. Histological depiction of the fourth inguinal mouse mammary gland and quantification of total mammary gland **a** area and **b** density on PND50 (*n* = 8–9/group). **c** Proliferation staining (Ki-67) quantification (percentage/1000 cells) with representative PND50 control and LP mammary gland sections (20× magnification, mean ± SEM, *n* = 6/group). **d** Morphological quantification (percentage/1000 cells) of apoptotic cells with representative PND50 control and LP mammary gland sections (100× magnification, mean ± SEM, *n* = 7–9/group). **e** Proliferation/apoptosis ratio in control and LP mammary tissue. Significant differences versus the control (CON) group were determined by *t* test. *P < 0.05, **P < 0.01. Scale bars= 3 mm (**a**,**b**) and 20 μm (**c**,**d**)

Given that the LP offspring had increased breast cancer risk, we then analyzed the effects of paternal suboptimal nutrition on their mammary development. We used postnatal day (PND)50 inguinal mammary glands to assess number of TEBs, ductal elongation, and epithelial area/density. Neither the number of TEBs (*P* = 0.96) nor the bodyweight-adjusted mammary ductal elongation (*P* = 0.97) was different between the groups. However, both total mammary epithelial area and epithelial density (*P* = 0.02 and *P* = 0.001, respectively; Fig. 2a, b) were significantly higher in LP offspring compared with controls.

Because we observed an increase in epithelial area as well as density in the mammary tissue of LP offspring, we tested whether this phenotype was due to a change in the balance between cell proliferation and apoptosis. Our analyses revealed that normal mammary tissue from LP offspring had lower rates of both proliferation (ki67 staining, *P* = 0.02; Fig. 2c) and apoptosis (morphologic assessment, *P* = 0.05; Fig. 2d) compared with controls. Although it was lower in LP mammary tissue, there were no statistically significant differences in the ratio of mammary proliferation/apoptosis between the groups (*P* = 0.13; Fig. 2e).

**Fig. 3:**
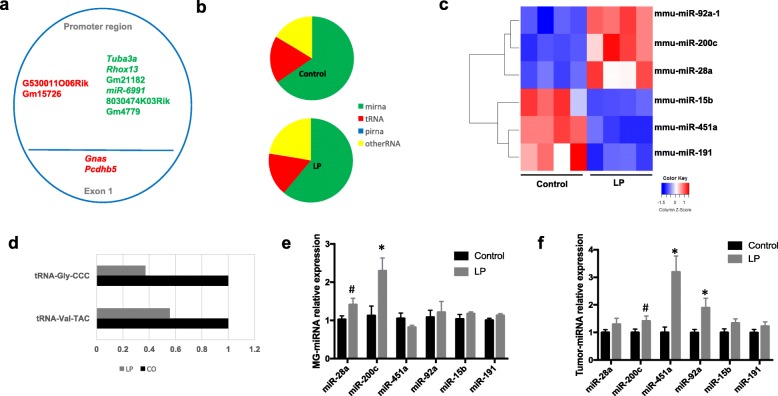
Paternal low-protein (LP) diet programs the mammary gland epigenome of female offspring. **a** Differentially methylated genes in PND50 control and LP mammary glands (green, hypomethylated; red, hypermethylated; *n* = 3/group) assessed by MBD-seq. **b** Pie chart showing the percentage of small RNAs mapped to specific subtypes in in PND50 control and LP mammary glands (*n* = 4/group) assessed by RNA-seq. **c** Heatmap showing differentially expressed miRNAs in PND50 control and LP mammary glands. **d** Fold-change of differentially expressed tRNA-derived fragments (tRFs) in PND50 LP mammary glands compared with controls. **e**,**f** Validation of differentially expressed miRNAs in control and LP mammary glands (*n* = 7–10/group) and tumors (*n* = 12/group) (mean ± SEM) by qPCR. Significant differences versus the control group were determined by *t* test. *P < 0.05, ^#^*P* < 0.07

It has been shown that ancestral paternal exposures are associated with epigenetic alterations in offspring [5, 9, 31]. Our genome-wide methylation analyses revealed few changes in mammary DNA methylation patterns in the promoter/exon 1 regions (Fig. 3a). Furthermore, we were unable to detect any meaningful changes in expression levels of the differentially methylated genes or in DNA methyltransferase genes using qPCR (Additional file 1: Table S5). However, using RNA-seq analyses, we found differences in the noncoding small RNA content between control and LP mammary glands. This analysis showed that the majority of small noncoding RNAs in mammary tissue were miRNAs, followed by tRFs, and other small RNA subtypes (Fig. 3b). A total of six miRNAs (upregulated: miR-28a, miR-92a, miR-200c; downregulated: miR-451a, miR-191, miR-15b) and two tRFs (downregulated: tRF5-Gly-CCC, tRF5-Val-TAC) were differentially expressed in mammary tissues of LP offspring compared with controls (Fig. 3c, d and Additional file 1: Table S4).

Given that miRNAs were the most abundant small RNA biotype in mammary tissue and, given their reported role in breast cancer [32], we focused on these molecules and downstream pathways for the remaining experiments. Validation of miRNAs by qPCR showed patterns of expression similar to those detected by RNA-seq in LP mammary tissues, with two exceptions: miR-191 and miR-15b. However, borderline or significant differences were only found for miR-28a (*P* = 0.06) and miR-200c (*P* = 0.01) (Fig. 3e).

In LP mammary tumors, miR-451a levels had a threefold increase compared with controls (*P* = 0.002). This increase was in contrast with normal LP mammary tissue where its levels were reduced. Both mir-92a1 (*P* = 0.02) and mir-200c (*P* = 0.07) also had higher expression in LP mammary tumors than in controls (Fig. 3f). miRNA expression can be regulated by DNA methylation [33]; however, integration of the genome-wide DNA methylation and RNA-seq data in control and LP normal mammary tissues did not reveal any statistically significant changes in DNA methylation patterns in promoter regions of differentially expressed miRNAs.

### Mammary tissues and tumors of LP daughters show altered amino-acid metabolism

Low birthweight and protein restriction have been shown to affect amino acid availability and metabolism [34–36]. Accordingly, a gene set enrichment analysis using the predicted target genes of the differentially expressed miRNAs showed that the top associated bio-function is amino-acid metabolism. A complete list of functions uncovered in our analyses is shown in Additional file 1 (Table S6).

**Fig. 4:**
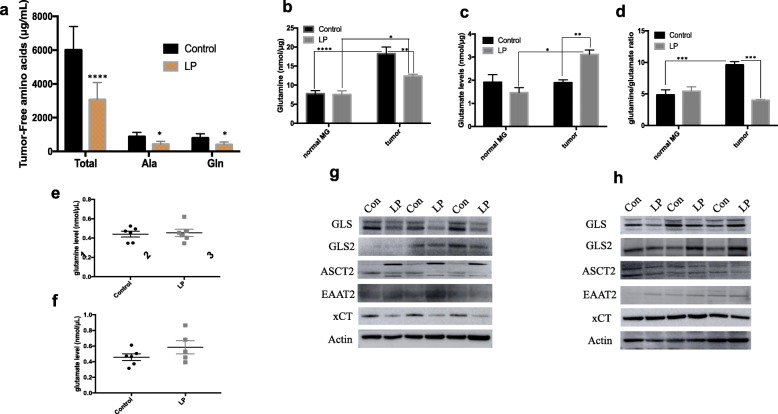
Amino acid metabolism is altered in low-protein (LP) diet mammary tissues and tumors. **a** Levels of free amino acids (total, alanine (Ala), and glutamine (Gln)) in control and LP tumors assessed by LC/MS (mean ± SEM; *n* = 6/group). **b**–**f** Levels of glutamine and glutamate in control and LP mammary glands and tumors (**b**-**d**), and in circulation (**e**-**f**) assessed by a colorimetric assay (mean ± SEM; *n* = 5–7/group). **g**, **h** Representative Western blots for GLS, GLS2, ASCT2, EEAT2, and xCT on PND50 control (Con) and LP mammary tissues (**g**) (*n* = 4–7/group) and tumors (**h**) (*n* = 5/group). Protein levels were normalized by β-actin. Significant differences versus the control group were determined as follows: two-way ANOVA (total amino acid levels, glutamine/glutamate in normal mammary tissue and tumors) and *t* test (glutamine/glutamate circulation). *P < 0.05, **P < 0.01***P < 0.001, ****P < 0.0001

Using a LC/MS analysis, we found that total free amino acid levels were significantly lower in LP tumors compared with controls (*P* < 0.0001). This reduction was mainly due to two amino acids, glutamine and alanine, which were significantly lower in LP tumors compared with controls (*P* < 0.05; Fig. 4a). Glutamine is one of the most abundant amino acids in the circulation and in cancer cells is known to be metabolized as a source of energy [37, 38]. Using a colorimetric assay, we further confirmed levels of glutamine and its metabolite, glutamate, using cell lysates from tumors. We found that glutamine levels were significantly lower and glutamate levels significantly higher, respectively, in LP tumors (*P* = 0.01 and *P* < 0.001) compared with controls. An assessment of glutamine and glutamate in LP mammary tissue lysates and plasma showed that levels of these amino acids were unchanged compared with controls. Interestingly, intratumor levels of glutamine were higher regardless of group while intratumor levels of glutamate were only in higher in the LP group compared with normal mammary glands (Fig. 4b–f).

Glutamine is metabolized to glutamate by glutaminases (GLS and GLS-2) and are carried in and out of the cell through several transporters [37, 39]. We therefore assessed the expression of these proteins using Western blot. Surprisingly, we found that levels of GLS were decreased while GLS-2 levels were unchanged in both LP mammary tissue and tumors in comparison with controls. We also found that levels of the glutamate transporter EEAT2 were unchanged in LP mammary gland and LP tumors. However, the glycosylated form of the glutamine transporter, ASCT2, was higher in LP mammary tissue, but not in LP tumors compared with controls. Levels of the glutamate-cystine antiporter xCT (Slc7a11) were decreased in LP mammary tissue. Compared with normal mammary tissue, LP tumors had increased expression of xCT, but not compared with control tumors (Fig. 4g, h).

### Mammary tissues and tumors of LP daughters have rewired nutrient-sensing mechanism

**Fig. 5:**
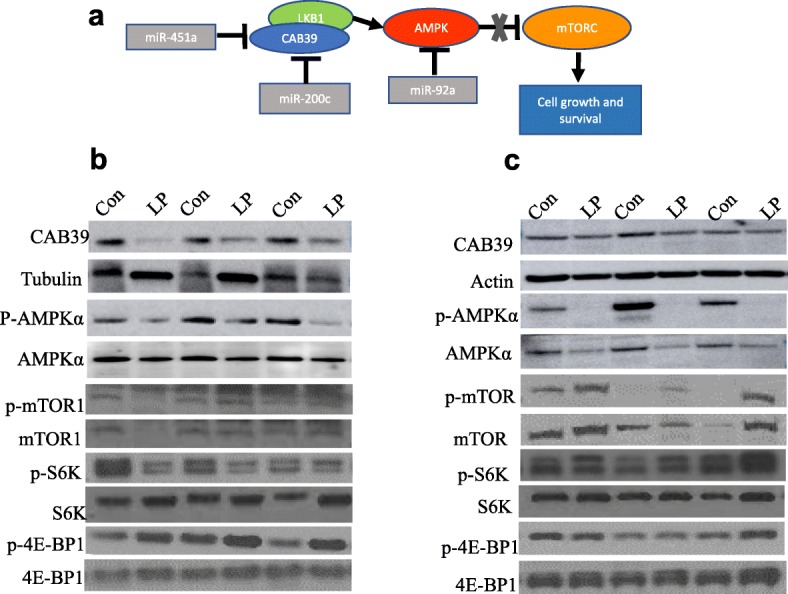
The AMPK signaling pathway is suppressed in low-protein (LP) diet mammary tissues and tumors. **a** Schematic representation of the interaction between miR-200c, miR-451a, and miR-92a1 with members of the AMP-activated protein kinase (AMPK) pathway. **b**,**c** Representative Western blots for CAB39, phospho-AMPK, AMPK, phospho-mTOR1, and mTOR1 on PND50 control (Con) and LP mammary tissues (**b**) (*n* = 4–7/group) and tumors (**c**) (*n* = 5/group). CAB39 protein levels were normalized by β-actin or β-tubulin. Phosphorylated proteins were normalized using the respective total protein levels

MicroRNAs 451a, 92a1, and 200c also regulate genes involved in other nutrient and energy sensing pathways. Both miR-451a and miR-200c are predicted or experimentally shown [40] to target *Cab39* mRNA, a component of the LKB1 complex which phosphorylates and activates AMPK. Furthermore, miR-92a is predicted to directly target the AMPK catalytic alpha subunit mRNA *Prkaa2*. Accordingly, we found that CAB39 expression was downregulated in LP tumors. We also found that both the expression and phosphorylation of AMPK was decreased in those tumors. In line with this, the levels of mammalian target of rapamycin (mTOR) activity and its downstream target S6 kinase were increased in LP mammary tumors. Interestingly, similar patterns of expression were detected LP mammary tissue, with reduction of CAB39 and AMPK levels compared with controls. While no increase in activity of mTOR or S6 kinase were observed in LP normal mammary tissues, an increase in 4E-BP1 phosphorylation was noted (Fig. 5).

### The expression of miRNAs and target genes altered in LP tumors differ by race/ethnicity and ER/PR/HER2 status in human breast tumors

**Fig. 6:**
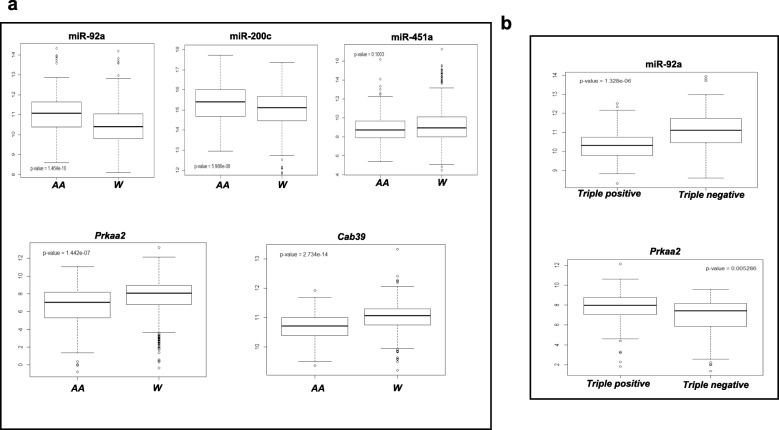
The miRNAs altered in LP tumors and downstream targets differ by race/ethnicity and tumor subtype in human breast tumors. **a** Box plot showing expression of miR-200c, miR-451a, miR-92a1, *Prkaa2*, and *Cab39* by race/ethnicity in breast tumors of TCGA database. **b** Box plot showing expression of miR-92a1 and *Prkaa2* by ER/PR/HER2 status in breast tumors of TCGA database. AA African American, W white

Low birthweight is prevalent in minority populations, with the highest rates observed in African-Americans [41], a group that is also afflicted with more aggressive breast cancers [42]. With that in mind, we asked whether the miRNAs (451a, 92a1, and 200c) differentially expressed in LP tumors varied by race/ethnicity and tumor subtype in humans. Using the TCGA database, we found that breast cancers in African-Americans have significantly higher expression of both miR-92a1 (*P* = 1.464 × 10^–10^) and miR-200c (*P* = 5.986 × 10^–8^), but not miR-451a, compared with whites. Furthermore, the expression of both *Cab39* (*P* = 2.734 × 10^–14^) and *Prkaa2* (AMPK catalytic alpha subunit; *P* = 1.442 × 10^–7^) is lower in breast tumors of African-Americans compared with whites. We also found that miR-92a1 expression is upregulated (*P* = 1.328 × 10^–6^) while the expression of its target gene, *Prkaa2*, is downregulated (*P* = 0.0053) in triple-negative breast cancers compared with triple-positive breast tumors (Fig. 6).

## Discussion

Using a mouse model, we found that paternal malnutrition (low-protein intake) led to reduced birthweight, alterations in mammary development, and increased mammary cancer risk in their female offspring. This phenotype was associated with differential expression of miRNAs (miR-200c, miR-92a, and miR-451a) known to regulate the AMPK energy-sensing pathway. Furthermore, we showed that the increase in mammary cancer risk in LP female offspring is associated with amino acid metabolism alterations, particularly with an increase in the energy-generating substrate glutamate.

Reports in the literature show that maternal protein restriction in gestation and lactation are associated with decreased birthweight, catch-up growth, and increased breast cancer risk in offspring [30]. We found similar results stemming from preconception paternal protein restriction. These, as well as our previously published findings, suggest that paternal exposures may be as important as maternal ones regarding determination of birthweight and cancer risk in offspring. As noted by others [43], there is a remarkable overlap between maternal and paternal exposure effects on offspring phenotypes, and our findings support this notion.

Epidemiologic studies have consistently shown that both high and low birthweight increases a woman’s breast cancer risk [11–13]. It is often assumed in those studies that birthweight is determined by maternal factors in pregnancy. Our data suggest that birthweight associated with paternal diet can also modulate breast cancer risk in offspring. Population studies have not directly investigated the link between paternal malnutrition and breast cancer, but indirect evidence exists. An analysis using the Dutch Famine Cohort has shown that women who were conceived during a famine, but not those exposed in later gestation, have increased breast cancer risk [18]. Furthermore, the association between other paternal environmental exposures and cancer risk in the offspring has been examined. For instance, paternal ethnicity and paternal age have been linked to the risk of breast cancer and hematological malignancies in their children [15, 16, 44]. In animal models, paternal overweight increases breast cancer risk in offspring [5]. Paternal dietary intake also modulates cancer risk, with both micronutrient deficiency and high saturated fat consumption increasing breast cancer risk in offspring [6, 45]. In addition to cancer risk, studies suggest that paternal exposures affect other diseases in the offspring, and sometimes grand-offspring, in both animals and humans [2, 9, 31, 46, 47].

Although we detected epigenetic changes (noncoding RNAs, DNA methylation) in LP male sperm, we did not directly investigate the mechanisms by which ancestral undernutrition information is transmitted to the offspring and modulates their cancer risk. However, recent published studies may offer some clues. Watkins and colleagues [4] report that paternal protein restriction is associated with reduced placental weight and alterations in placental nutrient transporters. Other studies suggest that sperm small noncoding RNAs can be delivered to the oocyte during fertilization and directly regulate gene expression in the developing embryo [7, 8]. Using zygote microinjections, Rodgers and colleagues [2] showed that miRNAs altered by paternal stress recapitulate the offspring stress phenotype in a mouse model. More recently, it was shown that tRFs, which are abundant in sperm, can also carry ancestral exposure memory from fathers to offspring. Others found that acquired DNA methylations patterns can be transmitted from fathers to offspring [33].

The extent by which protein restriction-induced changes in the male germline influence mammary gland development in their female progeny is also currently unknown. Our data, however, suggest that ancestral paternal protein intake is associated with changes in adult offspring mammary tissue, making this organ more prone to cancer development. The underlying rewiring of metabolism and energy-sensing mechanisms in LP mammary tissue may be partly responsible for the increase in breast cancer risk. Whether other systemic alterations in LP daughters, in addition to local mammary tissue changes, are important in determining that phenotype needs to be further explored. Previous studies report [3, 31], however, that ancestral diet can lead to metabolic abnormalities in the offspring suggesting that systemic changes may also be important. Of particular interest are reports showing that plasma amino acid levels are altered in adults who had low birthweight compared with those with normal birthweight, particularly when challenged with a high-calorie diet [36].

MicroRNAs regulate mammary tissue development [48] and tumorigenesis [32] and can act as either tumor suppressors or as oncomiRs depending on their target genes [49]. While functional studies are needed, published reports [40, 50] support the notion that miRNAs differentially expressed in mammary tumors of LP daughters target the tumor suppressive and energy-sensing AMPK pathway resulting in sustained mTOR activity and cell growth. Other alterations in nutrient metabolism were observed in LP daughters. We found that their tumors have reduced levels of total free amino acids, particularly alanine and glutamine. However, while reduced in LP tumors, the levels of glutamine are still higher compared with normal mammary tissue. This suggests that more glutamine, and possibly other amino acids, are in a bound state in LP tumor cells due to increased protein translation and cell growth. LP tumors also produce large amounts of glutamate which infers that more glutamine is being metabolized to glutamate in those tumors. Surprisingly, the levels of glutaminases were reduced (GLS) or unchanged (GLS-2) in LP mammary tumors compared with controls. This, however, is in line with inhibition of GLS expression by excess glutamate as well as protein restriction [37, 51]. Oncogenic alterations can render cancer cells addicted to glutamine, with rapidly dividing cells avidly consuming this amino acid [37]. Glutamate generated from glutamine can enter the tricarboxylic acid (TCA) cycle to produce energy and to act as a source of carbon and nitrogen to support nucleotides, fatty acids, and amino acid biosynthesis [37, 38]. Increased glutamine uptake and metabolism can further activate the mTOR pathway [52]. Together, these metabolic alterations may contribute to the higher growth rates seen in LP tumors, likely allowing cancer cells to withstand nutrient fluctuations under harsh conditions.

Our study also suggests that breast cancer phenotypes could be determined by an individual’s ancestral exposures. Our TCGA analysis and published findings [53] support this notion; breast tumors of women in ethnic groups with high rates of low birthweight show miRNAs and AMPK pathway alterations similar to those found in our animal model. Furthermore, it has been reported that more aggressive breast cancer subtypes such as triple-negative cancers are dependent on glutamine/glutamate metabolism and tend to have a low glutamine/glutamate ratio compared with ER-positive cancers [54]. Paternal protein restriction has been shown to perturb AMPK signaling in embryos, potentially as an adaptive mechanism to preserve viability [4]. Our study suggests that programming of the AMPK pathway and amino acid metabolism by ancestral LP diet persists through adulthood and, while it can be adaptive, it can also provide a growth advantage to transformed cells.

## Conclusions

In summary, we report that preconception paternal protein restriction can influence the birthweight, mammary gland development, and breast cancer risk and metabolism of daughters. Our findings could have important implications if confirmed in humans. First, our data support the notion that the heritable aspect of cancer risk and phenotype go beyond genetic factors. It could also result from ancestral exposures and possibly be inherited through modifiers of gene expression such as noncoding RNAs that could modulate organ development. Whether the miRNAs identified in our study are functionally responsible for increasing breast cancer risk in LP daughters still remains to be determined. However, our data strongly suggest that they may play a role in regulating tumor metabolism. Our findings could also help explain differences in breast cancer phenotypes and outcomes given that aggressive breast cancers with poor prognosis have rewired glutamine metabolism, a trait displayed by LP tumors. Finally, our results indicate that the effects of malnutrition on cancer risk go past the directly exposed individual. In humans, malnutrition and low birthweight are more prevalent in minority populations [41, 55]. Breast cancers in those populations are often more aggressive and have poorer prognosis [42]. Ancestral dietary patterns and lifestyle likely play a role in determining the breast cancer risk and outcomes in minority and low-income women, and needs to be further investigated in epidemiologic studies.

## Additional file

Additional file 1: **Figure S1.** Experimental design. a Male mice were fed the experimental diets (control or low protein (LP)) from 3 to 10 weeks of age. b Control and LP-fed males (F0) were mated to control-fed females to generate the offspring (F1) as shown. **Figure S2.** Representative results of *Actb* (a somatic cell marker) and *Smcp* (a sperm-specific marker) gene expression patterns by qRT-PCR in purified sperm samples after treatment with somatic cell lysis buffer. **Figure S3.** a Longitudinal bodyweight of males consuming a control or low-protein (LP) diet. b Diagram showing differentially methylated genes in control and LP paternal sperm assessed by MBD-seq. c Scatter plot showing small RNA abundance (counts per million) from control (*x* axis) and LP sperm (*y* axis) for different small RNA subtypes assessed by RNA-seq. d Pie chart showing the percentage of small RNAs mapped to specific subtypes in control and LP sperm. e Heatmap showing differentially expressed miRNAs in control and LP sperm. f Fold-change of differentially expressed tRFs in control and LP sperm. **Figure S4.** Gender-specific distribution in litters from LP and control fathers. **Table S1.** Composition of experimental diets. **Table S2.** Number of contributing fathers and female offspring per experiment. **Table S3.** List of antibodies used. **Table S4.** List of differentially expressed noncoding RNAs in LP fathers’ sperm and LP daughters’ mammary tissue. **Table S5.** Gene expression levels verified by qRT-PCR. **Table S6.** Biofunctions regulated by the microRNAs differentially expressed in LP daughters. (DOCX 1018 kb)
