# Supplementary material for: Paternal malnutrition programs breast cancer risk and tumor metabolism in offspring
Source: Breast Cancer Res. 2018 Aug 30;20:99. doi: 10.1186/s13058-018-1034-7 (PMC6117960; doi:10.1186/s13058-018-1034-7)
Supplement: Supplementary file 1 — Figure S1. Experimental design. a Male mice were fed the experimental diets (control or low protein (LP)) from 3 to 10 weeks of age. b Control and LP-fed males (F0) were mated to control-fed females to generate the offspring (F1) as shown. Figure S2. Representative results of Actb (a somatic cell marker) and Smcp (a sperm-specific marker) gene expression patterns by qRT-PCR in purified sperm samples after treatment with somatic cell lysis buffer. Figure S3. a Longitudinal bodyweight of males consuming a control or low-protein (LP) diet. b Diagram showing differentially methylated genes in control and LP paternal sperm assessed by MBD-seq. c Scatter plot showing small RNA abundance (counts per million) from control (x axis) and LP sperm (y axis) for different small RNA subtypes assessed by RNA-seq. d Pie chart showing the percentage of small RNAs mapped to specific subtypes in control and LP sperm. e Heatmap showing differentially expressed miRNAs in control and LP sperm. f Fold-change of differentially expressed tRFs in control and LP sperm. Figure S4. Gender-specific distribution in litters from LP and control fathers. Table S1. Composition of experimental diets. Table S2. Number of contributing fathers and female offspring per experiment. Table S3. List of antibodies used. Table S4. List of differentially expressed noncoding RNAs in LP fathers’ sperm and LP daughters’ mammary tissue. Table S5. Gene expression levels verified by qRT-PCR. Table S6. Biofunctions regulated by the microRNAs differentially expressed in LP daughters. (DOCX 1018 kb) [file 13058_2018_1034_MOESM1_ESM.docx]

**SUPPLEMENTARY INFORMATION FOR:**

**Paternal malnutrition programs offspring’s breast cancer risk and tumor metabolism in a mouse model**

**Raquel Santana^1#^, Elissa J. Carney^1#^, Johan Clarke^1^, Hong Cao^1^, M. Idalia Cruz^1^, Carlos Benitez^1^, Lu Jin^1^, Yi Fu^2^, Zuolin Cheng^2^, Yue Wang^2^ and Sonia de Assis^1^***

^1^Department of Oncology, Lombardi Comprehensive Cancer Center, Georgetown University, Washington, DC, USA

^2^Department of Electrical and Computer Engineering, Virginia Polytechnic Institute and State University, Arlington, VA, USA.

**# Co- first authors**

***Corresponding author:** Sonia de Assis, Ph.D., 3970 Reservoir Road, NW, The Research Building, Room E410, Washington, DC 20057, USA Phone: (202) 687-9518, Fax: (202) 687-7505, Email: [deassiss@georgetown.edu](mailto:deassiss@georgetown.edu)

**Authors email addresses:**

**Raquel Santana: r**s1844@georgetown.edu**,**

**Elissa J. Carney:** Elissa.carney@gmail.com

**Johan Clarke:** [jc827@georgetown.edu](mailto:jc827@georgetown.edu)

**Hong Cao:** hc87@georgetown.edu

**M. Idalia Cruz:** cruzi@georgetown.edu

**Carlos Benitez:** benitezc@georgetown.edu

**Lu Jin:** [lj74@georgetown.edu](mailto:lj74@georgetown.edu)

**Yi Fu:** fydennis@gmail.com

**Zuolin Cheng** zuolin8@vt.edu

**Yue Wang** yuewang@vt.edu

1. **Supplementary Figures and Tables**

**Figure S1**. Experimental Design: **a,** Male mice were fed the experimental diets (control or low-protein, LP) from 3 to 10 weeks of age. **b,** Control and LP-fed males (F0) were mated to control-fed females to generate the offspring (F1) as shown.

**Figure S2.** Representative results of *Actb* (a somatic cell marker) and *Smcp* (a sperm-specific marker) gene expression patterns, by qRT-PCR, in purified sperm samples (n=3) after treatment with somatic cell lysis buffer.

**Figure S3. a,** Longitudinal body weight of males consuming a control or low protein (LP) diet (n=11/group); **b,** Diagram showing differentially methylated genes in control and LP paternal sperm (green, hypomethylated; red, hypermethylated, n=2-3/group) assessed by MBD-seq; **c,** Scatter plot showing small RNA abundance (counts per million) from control (x axis) and LP sperm (y axis) for different small RNA sub-types (n=4/group) assessed by RNA-seq; **d,** Pie chart showing the percentage of small RNAs mapped to specific sub-types in control and LP sperm; **e,** Heatmap showing differentially expressed miRNAs in control and LP sperm; **f,** Fold-change of differentially expressed tRFs in control and LP sperm.

**Figure S4.** Gender-specific distribution in litters from LP and control

fathers (n=8-9/group).

**Table S1.** Composition of experimental diets

| *Ingredients* | *g/kg* | |
| --- | --- | --- |
|  | **Control (CO)** | **Low Protein (LP)** |
| Casein | 200.000 | 100.000 |
| L-Cystine | 3.000 | 2.000 |
| Corn Starch | 397.386 | 496.586 |
| Maltodextrin | 132.000 | 132.000 |
| Sucrose | 100.000 | 108.000 |
| Corn Oil | 60.000 | 60.000 |
| Soybean Oil | 10.000 | 10.000 |
| Cellulose | 50.000 | 50.000 |
| Vitamin Mix | 10.000 | 10.000 |
|  |  |  |
| **Protein (%)** | 17.7 | 8.9 |
| **Carbohydrate (%)** | 60.1 | 69.0 |
| **Fat (%)** | 7.2 | 7.1 |
| **Kcal/g** | **3.8** | **3.8** |

Control (TD.08819) and LP (TD.130983) are AIN93G-based diets containing either 17.7%

or *8.9*% energy from protein. Diets were manufactured by Envigo Teklad Diets.

**Table S2.** Number of contributing fathers and female offspring per experiment.

| **Samples** | **Assay** | **Group** | **Number of contributing fathers** | **Number of female offspring** |
| --- | --- | --- | --- | --- |
| **Mammary Gland** | **Apoptosis** | **CO** | 2 | 7 |
|  |  | **LP** | 2 | 9 |
|  | **ELISA** | **CO** | 2 | 7 |
|  |  | **LP** | 2 | 5 |
|  | **Ki67** | **CO** | 2 | 6 |
|  |  | **LP** | 2 | 6 |
|  | **DNA Methylation** | **CO** | 2 | 3 |
|  |  | **LP** | 2 | 3 |
|  | **RNA-seq** | **CO** | 2 | 4 |
|  |  | **LP** | 2 | 4 |
|  | **qRT-PCR** | **CO** | 2 | 7 |
|  |  | **LP** | 2 | 10 |
|  | **M.G. Development** | **CO** | 3 | 8 |
|  |  | **LP** | 2 | 9 |
|  | **Western Blot** | **CO** | 4 | 7 |
|  |  | **LP** | 2 | 4 |
| **Mammary tumor** | **Apoptosis** | **CO** | 4 | 10 |
|  |  | **LP** | 5 | 10 |
|  | **ELISA** | **CO** | 4 | 5 |
|  |  | **LP** | 3 | 5 |
|  | **Ki67** | **CO** | 5 | 6 |
|  |  | **LP** | 5 | 6 |
|  | **LC-MS** | **CO** | 5 | 6 |
|  |  | **LP** | 4 | 6 |
|  | **qRT-PCR** | **CO** | 3 | 12 |
|  |  | **LP** | 4 | 12 |
|  | **Tumorigenesis** | **CO** | 7 | 29 |
|  |  | **LP** | 7 | 32 |
|  | **Western Blot** | **CO** | 3 | 5 |
|  |  | **LP** | 4 | 5 |
| **Plasma** | **ELISA** | **CO** | 4 | 6 |
|  |  | **LP** | 3 | 5 |

Number of contributing fathers and female offspring used in each experiment by tissue type and experimental group.

**Table S3 - List of Antibodies Used**

| **Antibody** | **Application** |  | **Dilution** |  | **Description** |  | **Supplier** |
| --- | --- | --- | --- | --- | --- | --- | --- |
| 4E-BP1 | Western Blot |  | 1:1000 |  | Rabbit monoclonal |  | Cell Signaling # 9644S |
| AMPKα | Western Blot |  | 1:1000 |  | Rabbit polyclonal |  | Cell Signaling #2532 |
| Anti-CAB39 | Western Blot |  | 1:1000 |  | Rabbit monoclonal |  | Abcam #ab108279 |
| Anti-Ki67 | IHC |  | 1:7 |  | Rabbit monoclonal |  | BIOCARE #CRM325 |
| ASCT2 | Western Blot |  | 1:1000 |  | Rabbit polyclonal |  | Thermo Fisher Scientific #PA5-50527 |
| EEAT2 (GLT-1) | Western Blot |  | 1:1000 |  | Rabbit monoclonal |  | Thermo Fisher Scientific #701988 |
| GLS | Western Blot |  | 1:1000 |  | Rabbit monoclonal |  | Thermo Fisher Scientific #701965 |
| GLS2 | Western Blot |  | 1:1000 |  | Rabbit polyclonal |  | Abcam #ab113509 |
| Goat anti-Mouse IgG, HRP | Western Blot |  | 1:1000 |  | Goat polyclonal |  | Invitrogen #A16072 |
| Goat anti-Rabbit IgG, HRP | Western Blot |  | 1:1000 |  | Goat polyclonal |  | Invitrogen #A16104 |
| mTOR | Western Blot |  | 1:1000 |  | Mouse monoclonal |  | Cell Signaling #2983 |
| p70 S6 Kinase | Western Blot |  | 1:1000 |  | Rabbit monoclonal |  | Cell Signaling # 2708S |
| Phospho-4E-BP1 (Thr37/46) | Western Blot |  | 1:1000 |  | Rabbit monoclonal |  | Cell Signaling # 2855S |
| Phospho-AMPKα (Thr172) | Western Blot |  | 1:1000 |  | Rabbit monoclonal |  | Cell Signaling #2535 |
| Phospho-mTOR (Ser2448) | Western Blot |  | 1:1000 |  | Rabbit polyclonal |  | Cell Signaling #2971 |
| Phospho-p70 S6 Kinase (Thr389) | Western Blot |  | 1:1000 |  | Rabbit monoclonal |  | Cell Signaling # 9234 |
| SCL7A11 (xCT) | Western Blot |  | 1:1000 |  | Rabbit monoclonal |  | Abcam #ab175186 |
| β-Actin | Western Blot |  | 1:10000 |  | Rabbit monoclonal |  | Cell Signaling #8457 |
| β-Tubulin | Western Blot |  | 1:1000 |  | Mouse monoclonal |  | Thermo Fisher Scientific #MA5-16308 |

Specification for antibodies used in western-blot and immunohistochemistry (IHC) experiments.

**Table S4.** List of differentially expressed non-coding RNAs in LP fathers’ sperm and LP daughters’ mammary tissue

| **Non-coding RNA type** | **miRNA** | | | **tRNA fragments(tRFs)** | | | **piRNAs** | | |
| --- | --- | --- | --- | --- | --- | --- | --- | --- | --- |
| **Fathers** | **Name** | **Log2 Fold-change** | | **Name** | | **Log2 Fold-change** | **Name** | **Log2 Fold-change** | |
|  | \| *mmu-mir-10b* \| \| --- \| | 1.52 | | *tRNA-Ile-TAT-1* | | 1.12 | *piR-422* | 3.16 | |
|  | *mmu-mir-10a* | 1.67 | | \| *tRNA-Arg-ACG-2* \| \| --- \| | | \| 0.98 \| \| --- \| | *piR-104208* | -2.03 | |
|  | *mmu-let-7d* | 1.65 | | \| *tRNA-SeC-TCA-1* \| \| --- \| | | 0.60 |  |  | |
|  | *mmu-let-7c-1* | 2.22 | | \| *tRNA-Pro-AGG-1* \| \| --- \| | | -1.29 |  |  | |
|  | *mmu-let-7c-2* | 2.14 | | *tRNA-Ser-CGA-1* | | -0.97 |  |  | |
|  | *mmu-mir-3535* | 3.17 | |  | |  |  |  | |
|  | *mmu-mir-690* | 3.65 | |  | |  |  |  | |
|  | *mmu-mir-182* | 1.92 | |  | |  |  |  | |
|  | *mmu-mir-30a* | -1.40 | |  | |  |  |  | |
|  | *mmu-miR-30a-5p* | -1.70 | |  | |  |  |  | |
|  | *mmu-miR-22-3p* | -1.62 | |  | |  |  |  | |
|  | *mmu-miR-1a-3p* | -1.96 | |  | |  |  |  | |
|  | *mmu-mir-103-2* | 1.55 | |  | |  |  |  | |
|  | *mmu-mir-465b-2* | -1.91 | |  | |  |  |  | |
|  | *mmu-mir-465b-1* | -1.84 | |  | |  |  |  | |
|  | mmu-miR-30d-5p | -1.47 | |  | |  |  |  | |
| **Non-coding RNA type** | **miRNA** | | | **tRNA fragments(tRFs)** | | | **piRNAs** | | |
| **Daughters** | **Name** | | **Log2 Fold-change** | **Name** | **Log2 Fold-change** | | **Name** | | **Log2 Fold-change** |
|  | *mmu-miR-92a-1* | | 0.96 | \| *tRNA-Val-TAC-6* \| \| --- \| | \| -0.73 \| \| --- \| | | *piR-1330* | | 1.57 |
|  | *mmu-miR-200c* | | 1.32 | \| *tRNA-Gly-CCC-2* \| \| --- \| | -0.91 | | *piR-973* | | 1.36 |
|  | *mmu- miR-28a* | | 1.76 | \|  \| \| --- \| |  | |  | |  |
|  | *mmu- miR-15b* | | -2.16 |  |  | |  | |  |
|  | *mmu- miR-451a* | | -1.12 |  |  | |  | |  |
|  | *mmu- miR-191* | | -1.03 |  |  | |  | |  |

Non-coding RNAs differentially expressed (q value<0.05) in LP fathers’ sperm and LP daughters’ mammary tissue compared to controls assessed by small RNA-seq.

**Table S5.** Gene expression levels verified by qRT-PCR

| **Organ** | **Gene** | **Fold Change of Control** |
| --- | --- | --- |
| Mammary Gland | ***Dnmt1***  ***Dnmt3a***  ***Dnmt3b***  ***Rhox13***  ***Tuba3a***  ***Gnas***  ***Pcdhb5*** | 1.27  1.11  1.26  No detectable expression  No detectable expression  0.9  1.59 |

qRT-PCR assessment of expression levels of DNA methyltransferases (*Dnmts*) and genes differentially methylated in LP mammary glands by MBD-seq. Green, hypomethylated; Red, hypermethylated. Predicted genes expression was not assessed.

| **Table S6**. Bio-functions regulated by the microRNAs differentially expressed in LP daughters. | |
| --- | --- |
| **Bio-function** | ***P*-value** |
| Amino Acid Metabolism | 1.03E-06-1.67E-02 |
| Molecular Transport | 1.03E-06-2.77E-02 |
| Small Molecule Biochemistry | 1.03E-06-2.77E-02 |
| Organismal Survival | 1.47E-06-7.51E-03 |
| Behavior | 1.67E-06-3.03E-02 |
| Cell Morphology | 2.26E-05-3.02E-02 |
| Cellular Assembly and Organization | 2.26E-05-3.02E-02 |
| Cellular Development | 2.26E-05-3.02E-02 |
| Cellular Function and Maintenance | 2.26E-05-3.02E-02 |
| Cellular Growth and Proliferation | 2.26E-05-3.02E-02 |
| Nervous System Development and Function | 2.26E-05-3.02E-02 |
| Tissue Development | 2.26E-05-3.02E-02 |
| Cell-To-Cell Signaling and Interaction | 2.95E-05-2.85E-02 |
| Gene Expression | 4.8E-05-7.84E-04 |
| Digestive System Development and Function | 1.1E-04-1.71E-02 |
| Hepatic System Development and Function | 1.1E-04-5.63E-03 |
| Organ Morphology | 1.1E-04-3.02E-02 |
| Embryonic Development | 1.19E-04-2.4E-02 |
| Cell Death and Survival | 2.36E-04-2.83E-02 |
| Tissue Morphology | 2.36E-04-2.83E-02 |
| Neurological Disease | 2.63E-04-2.83E-02 |
| Psychological Disorders | 3.02E-04-2.47E-02 |
| Hematological System Development and Function | 3.52E-04-3.02E-02 |
| Humoral Immune Response | 3.52E-04-1.5E-02 |
| Lymphoid Tissue Structure and Development | 3.52E-04-2.4E-02 |
| Organismal Development | 3.85E-04-3.02E-02 |
| Organismal Injury and Abnormalities | 3.85E-04-3.02E-02 |
| Lipid Metabolism | 5.69E-04-2.73E-02 |
| Cellular Movement | 6.02E-04-2.42E-02 |
| Connective Tissue Disorders | 8.82E-04-1.89E-02 |
| Developmental Disorder | 8.82E-04-2.22E-02 |
| Gastrointestinal Disease | 8.82E-04-2.67E-02 |
| Skeletal and Muscular Disorders | 8.82E-04-1.73E-02 |
| Hematopoiesis | 9.72E-04-3.02E-02 |
| Organ Development | 9.72E-04-2.84E-02 |
| Auditory Disease | 9.78E-04-3.02E-02 |
| Auditory and Vestibular System Development and Function | 9.78E-04-3.02E-02 |
| Cancer | 9.78E-04-2.42E-02 |
| Cellular Compromise | 9.78E-04-2.42E-02 |
| Connective Tissue Development and Function | 9.78E-04-2.04E-02 |
| Drug Metabolism | 9.78E-04-9.78E-04 |
| Hematological Disease | 9.78E-04-2.04E-02 |
| Immunological Disease | 9.78E-04-1.5E-02 |
| Skeletal and Muscular System Development and Function | 9.78E-04-2.84E-02 |
| Respiratory Disease | 2.53E-03-2.56E-02 |
| Inflammatory Response | 2.54E-03-2.34E-02 |
| Carbohydrate Metabolism | 2.87E-03-2.26E-02 |
| Cardiovascular Disease | 2.87E-03-3.02E-02 |
| Cardiovascular System Development and Function | 2.87E-03-2.71E-02 |
| Nucleic Acid Metabolism | 2.87E-03-2.87E-03 |
| Hereditary Disorder | 3.39E-03-1.41E-02 |
| Endocrine System Development and Function | 4.11E-03-2.84E-02 |
| Respiratory System Development and Function | 4.11E-03-4.11E-03 |
| Ophthalmic Disease | 4.17E-03-4.17E-03 |
| Tumor Morphology | 5E-03-1.35E-02 |
| Cell Cycle | 5.63E-03-1.81E-02 |
| Endocrine System Disorders | 6.14E-03-8.71E-03 |
| Cell-mediated Immune Response | 6.48E-03-2.34E-02 |
| Reproductive System Disease | 6.9E-03-9.19E-03 |
| RNA Post-Transcriptional Modification | 7.9E-03-1.93E-02 |
| Reproductive System Development and Function | 8.71E-03-2.04E-02 |
| Renal and Urological Disease | 9.19E-03-1.35E-02 |
| Visual System Development and Function | 9.19E-03-9.19E-03 |
| Energy Production | 1.21E-02-1.21E-02 |
| Hepatic System Disease | 1.29E-02-1.29E-02 |
| Immune Cell Trafficking | 1.75E-02-1.85E-02 |
| Organismal Functions | 1.85E-02-2.42E-02 |
| Dermatological Diseases and Conditions | 2.42E-02-2.42E-02 |
| Hair and Skin Development and Function | 2.67E-02-2.67E-02 |

The predicted target mRNAs were used to generate a list of bio-functions using Ingenuity Pathway Analysis (IPA).
